# Genetic Transformation of *Metroxylon sagu* (Rottb.) Cultures via *Agrobacterium*-Mediated and Particle Bombardment

**DOI:** 10.1155/2014/348140

**Published:** 2014-09-11

**Authors:** Evra Raunie Ibrahim, Md. Anowar Hossain, Hairul Azman Roslan

**Affiliations:** ^1^CRAUN Research Sdn. Bhd., 93055 Kuching, Sarawak, Malaysia; ^2^Genetic Engineering Laboratory, Department of Molecular Biology, Faculty of Resource Science and Technology, Universiti Malaysia Sarawak, 94300 Kota Samarahan, Sarawak, Malaysia; ^3^Department of Biochemistry and Molecular Biology, University of Rajshahi, Rajshahi 6205, Bangladesh

## Abstract

Sago palm (*Metroxylon sagu*) is a perennial plant native to Southeast Asia and exploited mainly for the starch content in its trunk. Genetic improvement of sago palm is extremely slow when compared to other annual starch crops. Urgent attention is needed to improve the sago palm planting material and can be achieved through nonconventional methods. We have previously developed a tissue culture method for sago palm, which is used to provide the planting materials and to develop a genetic transformation procedure. Here, we report the genetic transformation of sago embryonic callus derived from suspension culture using* Agrobacterium tumefaciens* and gene gun systems. The transformed embryoids cells were selected against Basta (concentration 10 to 30 mg/L). Evidence of foreign genes integration and function of the* bar* and* gus* genes were verified via gene specific PCR amplification, gus staining, and dot blot analysis. This study showed that the embryogenic callus was the most suitable material for transformation as compared to the fine callus, embryoid stage, and initiated shoots. The gene gun transformation showed higher transformation efficiency than the ones transformed using* Agrobacterium* when targets were bombarded once or twice using 280 psi of helium pressure at 6 to 8 cm distance.

## 1. Introduction

Sago palm (*Metroxylon sagu*) is one of the most important plants contributing to the local economy and grown commercially for starch and/or conversion to animal food or fuel in Malaysia, Indonesia, Philippines, and Papua New Guinea. Sago palm research has been under focus because of the increasing need to explore nontraditional sources of food and fuel. Sago palm has a long life cycle, with an average of 15 years. Due to the long flowering time of sago palm and low seed germination rate, there is no report of breeding programs for sago palm [1], thus requiring alternative means of propagation for sago palm. Successful micropropagation of sago palm leaf tissues via direct shoot formation has been reported by several researchers [2–4]. The development of sago palm tissue culture technique serves as a basis by which genetic transformation can be conducted.

Two of the most common methods for plant genetic transformations are the* Agrobacterium*-mediated and the direct particle bombardment method.* Agrobacterium tumefaciens* is a soil borne, gram-negative bacterium that has a unique characteristic to transfer part of its genome to infect, transform, and parasitize plants. It has the ability to penetrate into cells at a wound site, actively transferring and integrating stably its genetic materials into the plant chromosomes [5]. The transformation mechanism works well with dicotyledonous plants; however, monocotyledonous plants are recalcitrant towards gene transfer using* Agrobacterium*. It is now possible to transform monocots using* Agrobacterium*, namely, via tissue culture; nevertheless various factors needed to be considered that contribute towards a successful genetic transformation. Among the factors involved included the genotype of plants, types and age of tissues used, the* Agrobacterium* strains and binary vectors used, and the various tissue culture conditions [6]. The* Agrobacterium*-mediated transformation protocol and improvement have been conducted in several monocots such as in rice by studying the effect of wounding, the use of different type of explants, the effect of osmotic pressure, and other modified parameters [7–10]. In maize, the transformation frequency was increased by modifying the medium used and the addition of* Agrobacterium*-inhibiting agent to optimize the coculturing and resting period [11–13]. Apart from that, the use of freshly isolated immature embryo or embryogenic calli was also able to increase the transformation efficiency in maize [14, 15]. In wheat transformed with* Agrobacterium*, a desiccation treatment has been shown to improve genetic transformation [16]. Apart from that the use of superbinary vector facilitates the transformation of sorghum and forage grass [17, 18]. Meanwhile, in the palm family,* Agrobacterium*-mediated transformation was recently reported in oil palm by Dayang et al. [19].

Particle bombardment is a method where high density, subcellular sized particles are accelerated to high velocity to carry genetic materials into living cells. This transformation involves the use of plant tissues or cells bombarded with either gold or tungsten particles coated with the foreign DNA, which is then incorporated into the plant chromosomes. Subsequently, surviving cells can be regenerated from the transformed tissue [20–22]. One main advantage of particle bombardment is that the method is species-independent and therefore has been commonly used now in monocot transformations such as in rice and other cereals [23, 24]. The method is also an efficient way to obtain new cultivars with desired traits as reported in date palm [25] and oil palm [26, 27]. In oil palm transformation research, Parveez et al. [26] reported that gene transfer using particle bombardment can be applied to many different cells and tissues with optimization of procedure in the DNA delivery conditions and tissues used. To date, report of genetic transformation of sago palm has not been reported. Therefore, the aim of this study was to develop and optimize a methodology for efficient genetic transformation of sago palm. This paper reports the transformation of sago palm, with the use of* Agrobacterium*-mediated and particle bombardment techniques, of embryonic calli from suspension cultures.

## 2. Materials and Methods

### 2.1. *Agrobacterium* Culture and* Agrobacterium*-Mediated Transformation of Sago Palm Embryogenic Callus

*Agrobacterium tumefaciens* strain LBA4404 was transformed with plasmid pGSA1131 containing* chloramphenicol acetyltransferase* gene (*cat*) for bacterial selection, a plant selectable marker* phosphinothricin acetyl transferase* gene (*bar*), and *β*-*glucuronidase *gene (*gus*). The* Agrobacterium *was grown in Luria-Bertani media (pH 7.0) containing 50 mg/L rifampicin and 35 mg/L chloramphenicol.The sago cells used for the transformation were derived from four stages of the suspension culture (D0C, D0E, D1-D2, and D3), pretreated onto plasmolysis medium (PM) with and without acetosyringone for an hour followed by the sonication.

In the* Agrobacterium*-mediated transformation, the pretreated calli were transferred into flasks containing* Agrobacterium* suspension and agitated slowly for 2 hours. The calli were then blotted dry on sterile filter paper and transferred onto the PM media and left for overnight. The next day, the cultures were transferred onto HB media (for cell multiplication) and incubated for three days. The cultures were washed with HB liquid media supplemented with 50 mg/L carbenicillin and then cultured onto fresh HB media (Table 1).

After one month, the cultures were transferred onto HB media containing 40 mg/L Basta and subcultured onto fresh media every month until regeneration of new callus. New regenerated callus was cultured back onto HB media for propagation. The putative transformed regenerants that developed into embryogenic calli were stained for gus activity, while embryoids were selected for molecular analysis.

### 2.2. Particle Preparation and Transformation of Sago Palm Embryogenic Callus via Helios Gene Gun

pGSA1131 plasmid was extracted using a Midi/Maxi Plasmid Purification Kit (Qiagen). The gold particles were coated with plasmid (1 *μ*g/*μ*L) mixed with 0.05 M spermidine and 1 M CaCl_2_. The pellet was then washed with 100% ethanol and resuspended in 3 mL of freshly prepared 50 *μ*g/mL PVP-ethanol. The gold-PVP-ethanol suspension was then loaded into a Teflon tube for use in Helios gene gun (Biorad). Meanwhile, sago palm embryogenic callus (0.3–0.7 cm in size) from the suspension culture was cultured onto HB media containing 10% sugar and incubated for 2 hours. After 2 hours, the embryogenic callus was ready for bombardment.

### 2.3. Particle Delivery Using Helios Gene Gun

The particle bombardment was carried out in aseptic conditions to avoid contamination of the cultures. A pressure between 200 and 300 psi was used for the helium gas and the distance of the gene gun from the target was adjusted to between 3 to 10 cm. After the bombardment, the transformed cells were kept on plasmolysis media for 2 hours and transferred on HB media for 2 weeks for recuperation and regeneration process. Subsequently, the transformed cells were transferred to HB media containing 40 mg/L Basta for one month for selection of transformants. Subculturing was conducted every one-month interval until new calli were regenerated. New callus was then transferred onto HB media for propagation. The putative transformed regenerants of embryogenic calli were stained for gus, while embryoids were selected for molecular analysis.

### 2.4. Analysis of Transformants

#### 2.4.1. Genomic DNA Extraction

Extraction of callus genomic DNA was carried out using the Plant DNA Extraction Kit (Qiagen). Approximately 2 grams of D3 stage embryoids or initiated shoots was grinded with a mortar and pestle in liquid nitrogen until it became powdery. Once the DNA has been eluted, polymerase chain reaction (PCR) analysis was conducted to verify the presence of foreign genes.

#### 2.4.2. PCR Analysis

The* bar* and* gus* genes were confirmed via gene specific PCR. The primers used to detect the* bar* gene were denoted as Bar3-F (5′ATG AGC CCA GAA CGA CGC 3′) and Bar3-R (5′ ATC TCG GTG ACG GGC AGG 3′) and meanwhile for the* gus *genes were the Gus-e F (5′ CCC CAG ATG AAC ATG GCA TCG 3′) and Gus-e R (5′ GG ATC CCC ATC AAA GAG ATC GCT 3′). The PCR was conducted according to the following protocol: denaturing step at 95°C for 5 min followed by 30 cycles of 94°C for 1 min, annealing step at 62°C for 2 min, extension step at 72°C for 1 min, and a final extension at 72°C for 10 min. The PCR products were analyzed on 1% of agarose gel.

### 2.5. Dot Blot Analysis

Dot blot analysis was undertaken with the use of the DIG DNA Labeling and Detection Kit, Dig Easy Hyb, and DIG Wash and Block Buffer Sets from Roche. The plant DNA was immobilized on a positively charged nylon membrane (Roche) via baking before hybridization using* bar*- and* gus*-labeled probes.

## 3. Results and Discussion

### 3.1. Transformation of Sago Palm Embryogenic Callus via* Agrobacterium*

**Figure 1:**
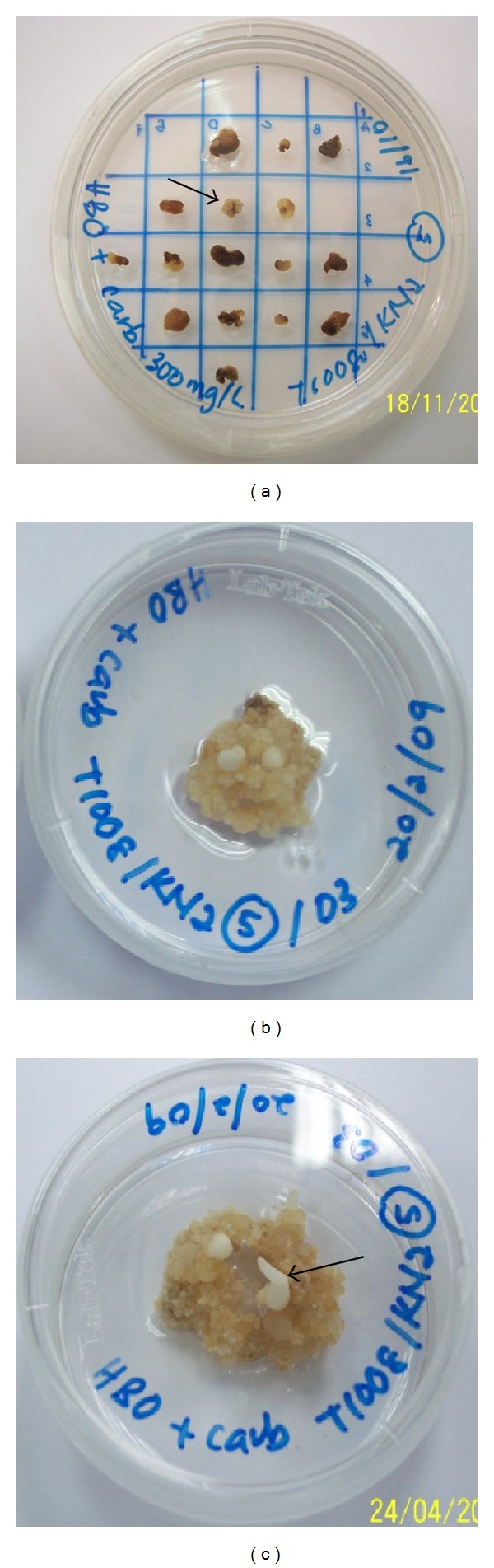
Development of callus after the* Agrobacterium*-mediated genetic transformation of sago palm. (a) Transformed embryogenic calli regenerated into new calli on Basta after 6 months of transformation. Putative callus selected for subculture is indicated by arrow. (b) Transformed callus after 9 months of transformation. Production of embryoids; (c) transformed callus after 10 months of transformation. The embryoids developing into initiated shoots (arrow).

**Figure 2:**
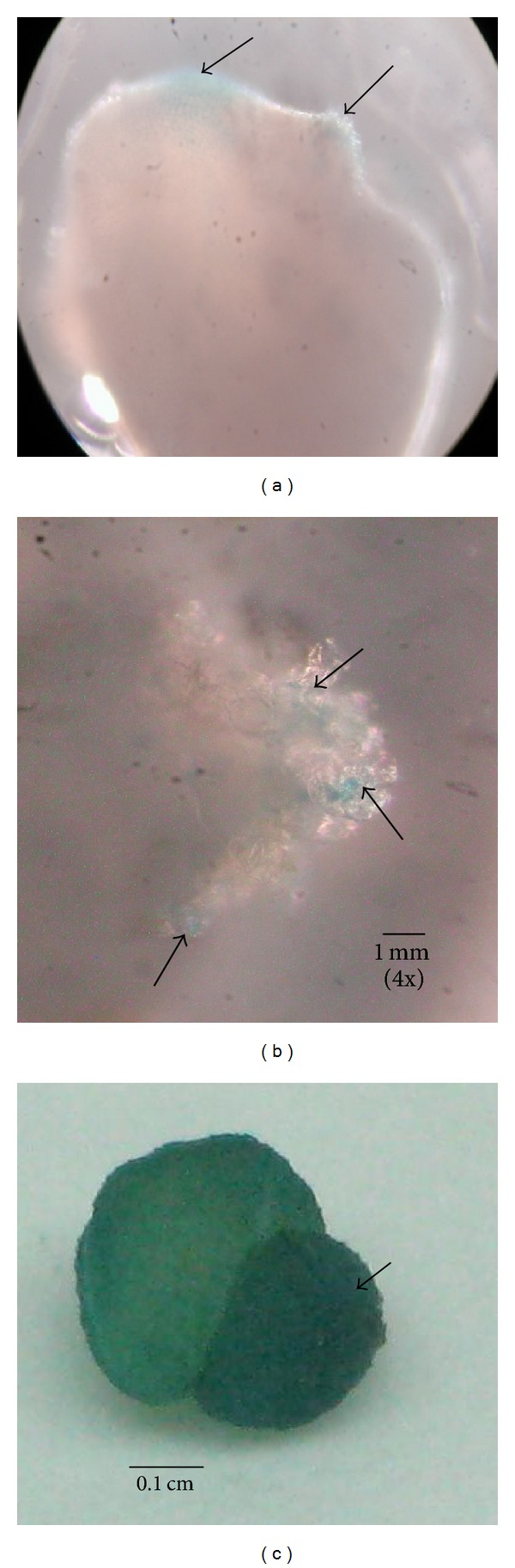
Gus histochemical staining of transformants at different stages of development. (a) 24 hours after* Agrobacterium* infection. (b) After 6 months and newly regenerated callus. (c) Transformed callus after 9 months producing embryogenic callus. Arrows indicate gus histochemical staining.

After cocultivation with* Agrobacterium*, the calli were subcultured on media containing 30 mg/L Basta and subsequently subcultured every 4 weeks. After one month, nontransformed callus showed sign of death, while transformed callus continued to grow on selection media. Transformed callus was separated into individual vessel and transferred onto fresh HB media without Basta selection where they multiplied and developed into initiated shoots (Figure 1). The results showed that transformed embryogenic calli regenerated into new calli on Basta after 6 months of transformation and the callus then produced embryoids after 9 months. Finally, the transformed embryoids developed into initiated shoots after 10 months of transformation. The embryogenic calli from five transformants showed blue coloration after gus histochemical staining, indicating the transfer and expression of* gus* gene in the genome of embryonic calli of sago palm (Figure 2).

### 3.2. Transformation of Sago Palm Embryogenic Callus via Helios Gene Gun

**Figure 3:**
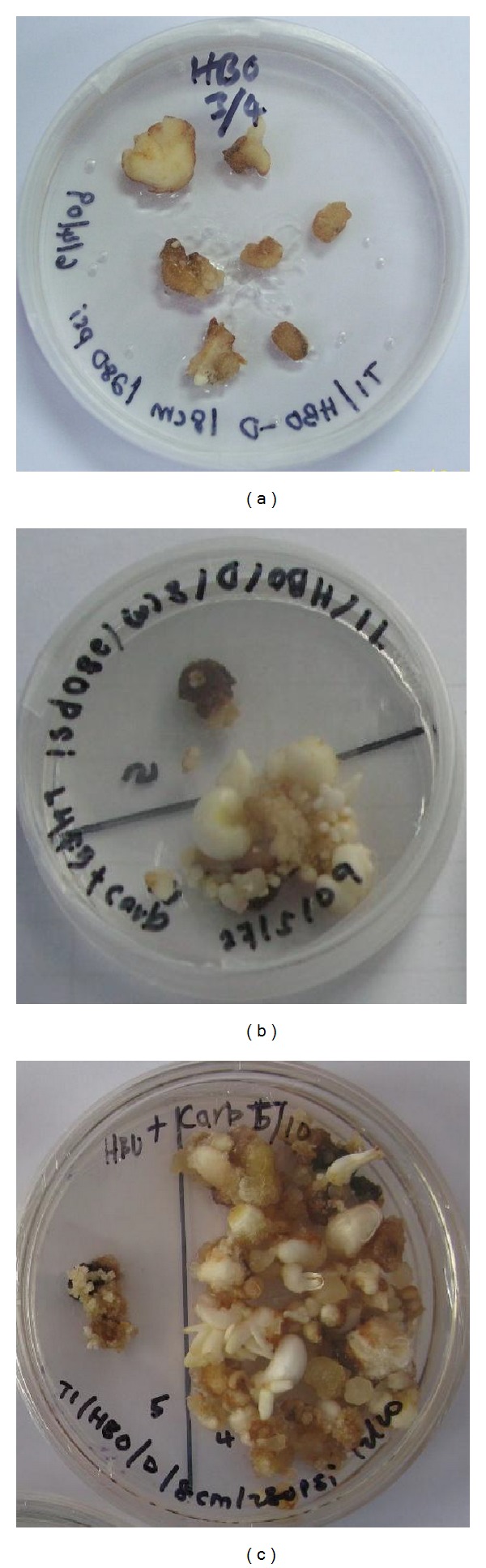
Development of transformants after gene gun transformation of sago palm. (a) Calli after 3 months of bombardment; (b) embryogenic callus that regenerates new calli after 6 months of transformation; (c) transformed embryogenic calli that regenerated successfully, developed into embryoids and initiated shoots.

**Figure 4:**
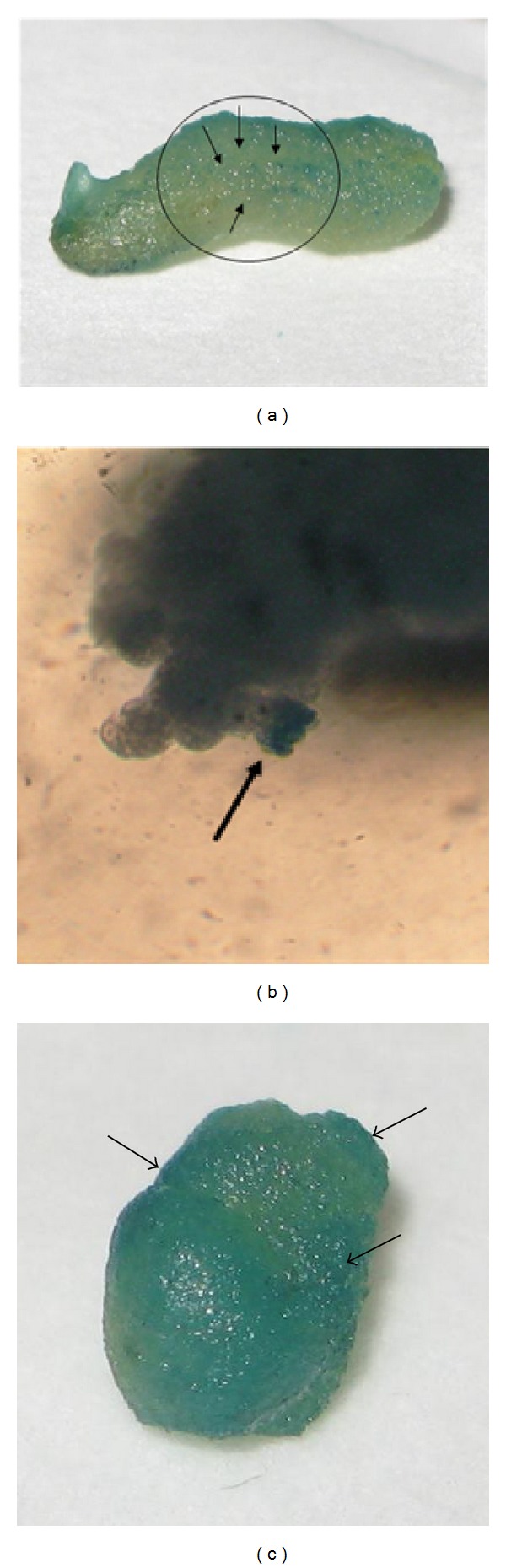
Gus histochemical staining of transformants transformed using gene gun at different stages of development. (a) 24 hours after bombardment; and arrows pointing at bombarded spots. (b) Callus regenerated after 3 months. (c) The transformed embryogenic calli after 9 months. Arrows indicate gus histochemical staining.

After the bombardment process, the callus was subcultured on media containing 30 mg/L Basta and subsequently subcultured on new media every 4 weeks. After one month, nontransformed callus showed sign of death, while twenty-four calli grew and showed resistance to selection. At this stage, the calli were transferred into fresh HB media individual plates without Basta; however, only seventeen had multiplied and developed into initiated shoots (Figure 3). The embryogenic calli from these transformants were subsequently analyzed with gus histochemical staining (Figure 4).

### 3.3. Optimization of Particle Bombardment Parameters

The transformants were mostly produced after the bombardment of sago embryogenic callus using 280 psi of helium pressure with a 6 cm distance of the gun to the target and with once (1x) or twice (2x) bombardment (Table 2). Helenius et al. [28] and Carsono and Yoshida [24] suggested that pressure of between 200 and 250 psi is the best to use with the distance of about 2-3 cm to intact plant cells. The helium pressure and the distance used in this sago palm embryonic callus transformation were slightly higher due to the different type of explants used. For particle bombardment, similar to the* Agrobacterium*-mediated transformation, the use of embryogenic suspension calli (D0E) was better as compared to the use of callus stage D0C and embryoid stages of D1, D2, and D3 (Table 2). Previous works on rice and oil palm successfully showed transformations with the use of immature embryos or embryogenic calli as explants [26, 29–31].

Another parameter analyzed is the number of bombardments conducted on the target cells, that is, once (1x), twice (2x), and thrice (3x). The site of each bombardment was different by rotating the target vessel 90° in between each bombardment to have a better coverage of the target area and increases the efficiency of transformation [25]. From our work, embryogenic callus bombarded 1x and 2x is able to survive the procedure. Janna et al. [32] also reported that there was no significant difference observed between 1x and 2x bombardments; however, 2x bombardments have been shown to increase the transformation efficiency in banana [33], Brazilian maize [34], and date palm [25].

### 3.4. Integration of Foreign DNA Analysis via Dot Blot

#### 3.4.1. Via* Agrobacterium*-Mediated Transformation

**Figure 5:**
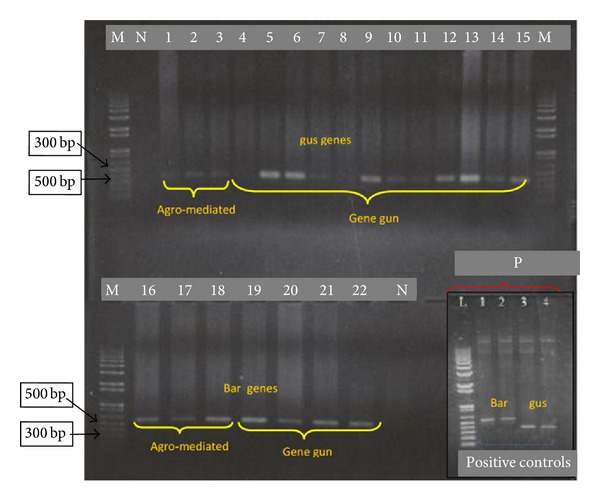
Agarose gel electrophoresis of PCR amplification products for twenty-two putative transformed and control samples from* Agrobacterium* and particle bombardment transformation methods. Lanes denoted as M represent the 1 kb DNA ladder (Fermentas); N represent the amplification of negative control (untransformed embryoids); section P represents the amplification of positive controls for* gus* and* bar* genes. Lanes 1–3 and 4–15 are amplification products for* gus* genes from* Agrobacterium*-mediated and particle bombardment methods, respectively. Meanwhile, lanes 16–18 and 19–22 are amplification products for* bar* genes from* Agrobacterium*-mediated and particle bombardment methods, respectively.

**Figure 6:**
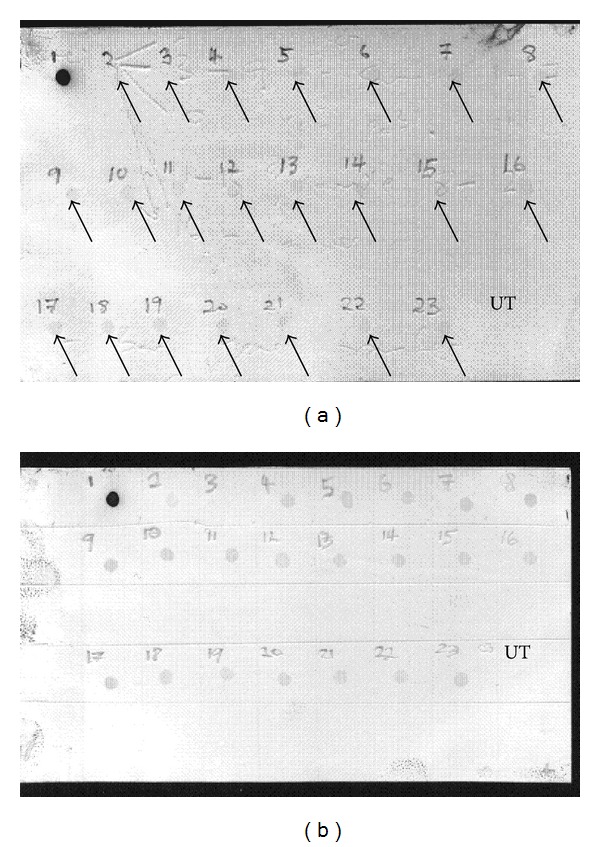
Dot blot analysis of transformants harboring* gus* and* bar* genes. (a) and (b) represent dot blot hybridization using* gus *and* bar *probes, respectively. Sample 1 represents the positive control. Samples 2–6 and 7–23 are DNA samples extracted from regenerated callus from* Agrobacterium*-mediated method and particle bombardment methods, respectively. UT denotes sample from the untransformed callus.

The genomic DNA was isolated from the transformed D3 embryoids and initiated shoots, and PCR amplification was carried out using gene specific primers for* gus* and* bar* genes. The PCR products were analyzed on 1% agarose gel electrophoresis (Figure 5). Lanes 1–3 (*gus* genes) and 16–18 (*bar* genes) showed the amplification products with the expected sizes and indicated the presence of both genes in the samples (Figure 5). Meanwhile, a dot blot analysis was also conducted to confirm the integration of* gus* and* bar* genes in transformed calli and initiated shoots samples (Figure 6).

#### 3.4.2. Via Helios Gene Gun Transformation

The genomic DNA was isolated from the transformed D3 embryoids and initiated shoots, and PCR amplification was carried out using gene specific primers for* gus* and* bar* genes. The PCR products were analyzed on 1% agarose gel electrophoresis. In Figure 5, lanes 4–15 (*gus* genes) and 19–22 (*bar* genes) represent the amplification products with the expected sizes and indicated the presence of both genes in the samples (Figure 5). A dot blot analysis was also conducted to confirm the integration of* gus* and* bar* genes via particle bombardment method in calli and initiated shoots samples (Figure 6). This study showed that both methods can be used to transform sago palm embryogenic calli from suspension culture and to regenerate new callus within a six-month period.

Hiei et al. [6] and Dayang et al. [19] previously reported the transformation of rice and oil palm using callus. This highly regenerative callus gives large number of transformed lines as observed in several monocots such as wheat [35] and rice [36]. Other than that, Cheng et al. [16] showed that wounding was not essential for T-DNA delivery certain monocot species. However, reports by Zuker et al. [37] and Dayang et al. [19] have shown that wounding the target samples assisted in the* Agrobacterium*-mediated transformation of oil palms.

The osmotic treatment of the target cells is generally practiced in the particle bombardment method for both monocot and dicot species. In our work, the sago palm cells that were regenerated on the Basta selection medium were treated with both 10% of sucrose and 0.55 M of mannitol compared to the 6% sucrose, which was the normal sucrose level for sago palm propagation. The results showed that the osmotic treatment assisted the transformation process with equal impact. On the other hand, Parveez et al. [26] and Mousavi et al. [25] found that 0.4 M mannitol gives higher transformation rate in oil palm and date palm, respectively. Meanwhile, the addition of acetosyringone to induce the transformation process was found not to have much impact on the transformation of sago palm cells (data not shown) despite the reports that stated that the addition of acetosyringone is recommended in the transformation of monocotyledonous plant [19, 38–40].

## 4. Conclusion

This work has determined that genetic transformation of sago palm cells is achieved via both* Agrobacterium*-mediated and particle bombardment systems. The transformation rate by particle bombardment was calculated to be 1.4% while by* Agrobacterium*-mediated bombardment was much lower at 0.5%. To determine the integration of the foreign DNA, dot blot analysis was conducted and showed that the* gus* and* bar* genes were present and integrated into sago palm genome. The embryogenic callus was also determined to be the most suitable explant to be used for the transformation process. We have shown that it is possible to introduce foreign genes into sago palm; nevertheless, more studies are required to further characterize the transformation.

## Figures and Tables

**Table 1:** Medium composition.

Medium	Composition
HB	MS salts (Murashige and Skoog) + 6% sucrose + 0.3 g/L inositol v5 + vitamins + 2 mg/L NAA + 2 mg/L 2,4-D + 0.1 g/L glutamine + 2.5 g/L Gelrite.
HB liquid	HB media without agar.
OSM	HB media with addition of 0.2 M acetosyringone; sucrose was increased to 10%.
CCM	HB liquid with addition of 0.2 M acetosyringone.
LB	LB-Lennox formula which contained 10 g tryptone, 5 g yeast extract, and 5 mg of NaCl in 1 litre.

**Table 2:** List of the conditions used in particle bombardment transformation of sago palm explants. The optimized condition includes the number of bombardments, helium pressure, distance of gene gun to targets, and media used for pretreatment of target cells. Twenty-four transformants were generated from four replicates of three trials. The transformants were selected on Basta and subsequently regenerated new callus.

Transformant number	Bombardment conditions				Type of target
	Number of bombardments	Helium pressure	Distance	Media	
1	1x	280 psi	8 cm	Plasmolysed	D3
2					**D**0E*
3					D0E
4					**D**2*
5					D0E
6					D0E

7	1x	280 psi	6 cm	Plasmolysed	D0E
8					**D**0E*
9					D0E
10					D0E
11					**D**2*
12					**D**0E*
13					D0E
14					D2
15					D0E
16					D0E
17					D0E

18	2x	280 psi	6 cm	Plasmolysed	D0E
19					D0E
20					D3

21	2x	280 psi	5 cm	Plasmolysed	D0E
22					D0E
23					D2
24					D2

*Transformants that could not produce embryogenic calli and embryoid bodies.
